# Paper-Based Microfluidic Chips for At-Home Point-of-Care Nucleic Acid Testing: Applications and Challenges

**DOI:** 10.3390/diagnostics16020251

**Published:** 2026-01-13

**Authors:** Hao Liu, Yuhan Jia, Yitong Jiang, You Nie, Rongzhang Hao

**Affiliations:** 1 Department of Toxicology and Sanitary Chemistry, School of Public Health, Capital Medical University, Beijing 100069, China; 6hhhhhh@mail.ccmu.edu.cn (H.L.); jiayh@mail.ccmu.edu.cn (Y.J.); jiangytong202412@163.com (Y.J.); 2 Beijing Key Laboratory of Environment and Aging, Beijing 100069, China; 3 Yanjing Medical College, Capital Medical University, Beijing 102300, China; 4 Laboratory of Respiratory Diseases, Beijing Pediatric Research Institute, Beijing Children’s Hospital, Capital Medical University, Beijing Key Laboratory of Core Technologies for the Prevention and Treatment of Emerging Infectious Diseases in Children, Beijing 100045, China; 5 Key Laboratory of Major Diseases in Children, Ministry of Education, National Clinical Research Center for Respiratory Diseases, National Center for Children’s Health, Beijing 100045, China

**Keywords:** paper-based microfluidics (μPADs), nucleic acid testing, point-of-care testing (POCT), infectious disease diagnostics

## Abstract

Along with the growing demands for personalized medicine and public health surveillance, diagnostic technologies capable of rapid and accurate pathogen nucleic acid testing in home settings are becoming increasingly crucial. Paper-based microfluidic chips (μPADs) have emerged as a potential core platform for enabling molecular testing at home, owing to their advantages of low cost, portability, and independence from complex instrumentation. However, significant challenges remain in the current μPADs systems regarding nucleic acid extraction efficiency, isothermal amplification stability, and signal readout standardization, which hinder their practical and large-scale application. This review systematically summarizes recent research progress in μPADs for home-based nucleic acid testing from four key aspects: extraction–amplification–detection system integration, with a particular focus on the synergistic effects and development trends of critical technologies such as material engineering, fluid control, signal transduction, and intelligent readout. We further analyze typical application cases of this technology in the rapid screening of infectious disease. Promising optimization pathways are proposed, focusing on standardized manufacturing, cold-chain-independent storage, and AI-assisted result interpretation, aiming to provide a feasible framework and forward-looking perspectives for constructing home-based molecular diagnostic systems.

## 1. Introduction

Nucleic acid testing (NAT) represents the gold standard for the identification of infectious pathogens due to its unparalleled sensitivity, specificity, and ability to detect early-stage or asymptomatic infections. In recent years, polymerase chain reaction (PCR) technology has developed rapidly, achieving significant improvements in reagent and instrument costs, detection sensitivity, and speed [1,2]. However, current PCR detection commonly implemented in hospitals still relies on high-speed thermal cycling precision instruments and professional personnel operation. In cases of daily respiratory infections, home-based detection can enable extremely rapid pathogen identification, thereby facilitating targeted treatment at early stages. For sexually transmitted infection (STI) diagnosis, which also requires necessary testing due to privacy concerns, there is an urgent need for detection methods. In public health emergencies, delays in centralized testing can lead to missed diagnoses during early infection stages, thereby hindering timely isolation and treatment. Some chronic infection patients require long-term pathogen load monitoring to evaluate treatment efficacy and infection progression, with repeated monitoring potentially leading to reduced compliance and unfavorable treatment outcomes [3,4]. These limitations highlight the urgent demand for point-of-care (POC) platforms deployable at home settings.

Paper-based microfluidics, as an emerging POC platform, requires no external pump sources, offers simple operation, and facilitates storage and transportation. In recent years, the proposed REASSURED principles have expanded requirements for affordability, ease of use, and equipment independence, inheriting ASSURED criteria while adding real-time connectivity and environmental sustainability dimensions, providing more comprehensive and cutting-edge guidance for modern diagnostic technology development, particularly in digitalization and intelligent directions [5,6]. Nucleic acid testing methods integrated with Paper-based microfluidics (μPADs) demonstrate significant application potential and development prospects. Their porous cellulose matrices can efficiently complete sample filtration, nucleic acid capture, and inhibitor removal, significantly simplifying nucleic acid extraction processes. Leveraging capillary action enables fluid control without external pump-driven power, automatically and sequentially completing multi-step reactions including lysis, amplification, and detection. The low cost and scalable manufacturing potential of paper materials make disposable use possible, greatly reducing detection barriers [7,8].

Emerging strategies are rapidly gaining attention. These strategies include three-dimensional origami architectures or nanomaterial-assisted signal amplification; integration of Clustered Regularly Interspaced Short Palindromic Repeats (CRISPR)-based detection modules to improve sensitivity and specificity [9,10,11]; and smartphone-compatible readout platforms to enable digital health connectivity. Additionally, increasing emphasis is being placed on scalable and sustainable manufacturing processes to ensure practical deployment in resource-limited environments [12,13]. In this review, we comprehensively overview recent advances in μPAD-based nucleic acid diagnostic technologies, with particular focus on structural engineering, integration of amplification technologies, signal readout strategies, and applications of paper-based microfluidics in home-based testing environments. We further emphasize current research hotspots and discuss the future prospects of transforming μPADs platforms into practical monitoring tools for home scenarios. We further emphasize current research hotspots and discuss the future prospects of transforming μPADs platforms into practical monitoring tools for home scenarios. While existing reviews have extensively discussed fabrication techniques, signal amplification strategies [14], and mobile readout integration [15], they have not adequately addressed how to balance the simplicity required for home users with the technical complexity needed for reliable nucleic acid detection. A key challenge remains in developing standardized protocols that can maintain performance across diverse home environments without imposing high user expertise requirements. Despite recent advances, gaps remain in the translation of laboratory-scale μPADs into mass-producible and commercially viable products suitable for real-world storage and handling.

## 2. Fabrication and Structural Engineering of μPADs

μPADs were first introduced by the Whitesides research group at Harvard University in 2007, utilizing wax printing technology to construct hydrophilic and hydrophobic channels on paper [16], thereby enabling passive liquid manipulation. μPADs can integrate sample separation, transport, and detection steps within a miniaturized volume, offering advantages including low cost, biocompatibility, liquid adsorption through capillary action without external power requirements, and ease of modification. Leveraging their platform-based and miniaturized design advantages, μPADs are suitable for rapid self-testing applications in home settings, such as infectious disease screening, chronic disease management, and reproductive health management [17]. As detection scenarios become increasingly complex, μPADs face higher requirements for sensing performance, channel structure, and reaction integration, with their sensitivity, reaction speed, and automation level requiring urgent improvement [17,18].

The demand for high-performance and high-integration field applications has placed higher requirements on μPADs fabrication techniques and structural engineering, which has become a key research direction [19]. In practical applications, μPADs platforms often integrate various functional materials according to different detection purposes, including paper substrates, polymer hydrophobic barriers, biological probes, or nano-enhancement components [20].

These materials exhibit diverse physical and chemical properties, requiring targeted fabrication methods to achieve coordinated functionality, precisely controlling channel structure, surface properties, and fluid behavior at millimeter and micrometer scales. To address the problems of manual sample addition and complex reaction steps in traditional μPADs, recent research has combined microfluidic chips with paper-based platforms, introducing origami structures and self-actuated paper valves as key components to construct novel fluid control platforms.

This section will examine fluid transport behavior on paper-based platforms and its regulatory mechanisms on reaction kinetics, common channel formation techniques, and structural design for paper-based microfluidic platforms.

### 2.1. Integration of Flow Control and Reaction Kinetics

Unlike traditional microfluidic systems, μPADs rely on capillary action within bulk-manufactured fibrous porous materials of all types for liquid driving, where capillary-driven fluid behavior directly determines reaction efficiency and consequently affects sensitivity. When liquid contacts paper material, it automatically migrates along pre-designed hydrophilic channels under capillary force, with flow velocity approximately estimated by the modified Lucas-Washburn theory (Equation (1)) [21,22].

(1) $$v(t)=\sqrt{\frac{\gamma \cdot \cos\theta \cdot r}{2\eta \cdot t\cdot \tau }}$$

Equation (1) Modified Lucas–Washburn model for capillary-driven liquid transport in paper-based porous media. where $v\left(t\right)$ is the liquid flow velocity, *γ* represents the liquid surface tension, *θ* is the contact angle between liquid and paper fiber, *r* is the effective pore radius of the porous paper network, *η* is the liquid viscosity, *t* is time, and *τ* represents the tortuosity factor, which accounts for the complex flow paths within the fiber matrix.

Factors influencing flow behavior include paper pore size and wettability, liquid viscosity, environmental humidity, and channel geometric parameters. The interaction between capillary action, chromatographic migration, and diffusion collectively determines reaction kinetics in μPADs. Regarding the influence of flow velocity on capillary action, Meng et al. experimentally found that the signal intensity difference between positive and negative sample detection lines significantly increased within the first 6 min, subsequently decreasing [23]. The signal evolution reveals a critical interplay between fluid residence time and the intrinsic reaction kinetics of the Pd@Ir nanozyme. The rapid signal rise within the initial 6 min is driven by the high catalytic turnover, where the residence time is sufficient for effective substrate conversion. Conversely, the signal plateau and subsequent decline suggest a transition to a diffusion-limited regime. This phenomenon indicates the existence of an optimal reaction time window, where insufficient time leads to incomplete reactions, while excessive time may cause signal reduction due to factors such as “hook effect”. Therefore, maintaining appropriate reaction residence time while ensuring rapid detection represents a core challenge in μPADs design [24,25].

**Figure 1:**
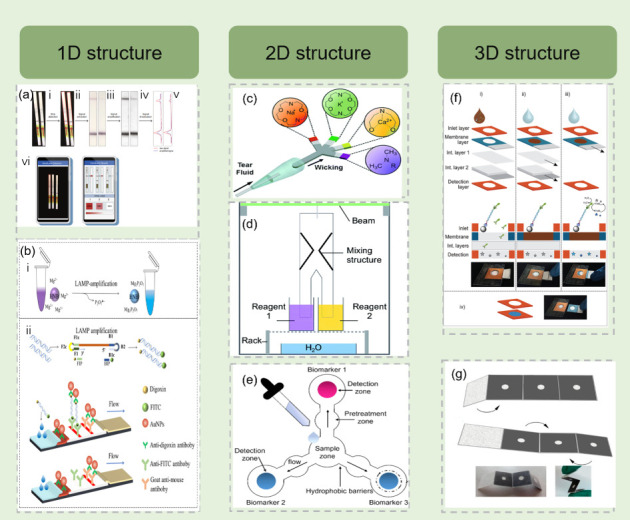
Representative structures of μPADs. (**a**) 1D linear strip for simple, unidirectional fluid transport in viral diagnostics. (**i**) Images of positive lateral flow strips are captured by the app. (**ii**) Signal regions are automatically identified. (**iii**) Image denoising and smoothing are applied. (**iv**) Image contrast is enhanced. (**v**) Signal profiles are extracted for analysis. (**vi**) The app processes strip images and outputs qualitative and semi-quantitative results [30]. (**b**) 1D device with defined zones for colorimetric pathogen detection. (**i**) Principle of the colorimetric LAMP assay. The reaction mixture initially appears purple due to the presence of HNB and Mg^2+^. During amplification, Mg^2+^ is consumed, resulting in a color change to blue, while negative reactions remain purple. (**ii**) Principle of the LAMP-ICT assay. LAMP products labeled with FITC and digoxin are captured on a lateral flow strip via gold-labeled antibodies, producing visible bands on the test and control lines for positive samples, while only the C line appears for negative samples [31]. (**c**) 2D planar network enabling fluid distribution for multiplexed analysis [32]. (**d**) 2D device with engineered patterns to enhance passive mixing [26]. (**e**) A branched 2D μPAD with a central inlet branching into three independent reaction zones for the simultaneous colorimetric detection of multiple salivary biomarkers [33]. (**f**) 3D stacked-layer architecture for complex enzyme-linked assays. (**i**) add bead complexes, (**ii**) add washing buffer, (**iii**) add H_2_O_2_ and incubation, (**iv**) readout [34]. (**g**) Schematic of an all-in-one origami paper-based device (oPAD) integrates cell lysis, protein extraction, and DNAzyme-mediated signal amplification into a single foldable platform, enabling instrument-free readout.

Concentration gradient-driven diffusion serves as the primary mechanism for reactant mixing within paper substrates, occurring simultaneously between channels and flow paths. Hamidon et al. utilized hourglass-shaped channels to enhance signal intensity. According to the underlying theory, narrowing the gap width of from 4.69 mm to 0.32 mm serves to effectively handle the dynamic range of the concentration gradient generated after the mixing step. This geometric constriction spatially compresses the analyte distribution, thereby resulting in a more concentrated and uniform signal readout (Figure 1a). However, diffusion rates require precise balancing: too slow leads to reaction delays, while too fast may cause analyte dilution or even loss [26]. Ayushi Chauhan and Tooley developed a barrier-free design capable of achieving rapid distribution across 20 channels, effectively avoiding colorimetric detection errors caused by slow flow rates and incomplete mixing, completing all detection within 30 s [27]. Additionally, large-pore filter paper materials such as Whatman Grade 1 have been widely used for rapid reaction systems, with their high porosity helping to reduce liquid transport time, suitable for detection scenarios requiring quick completion [28]. In contrast, while slower flow rates can ensure sufficient reaction completion, they extend detection time. Batule et al. developed a μPAD-RT-LAMP (reverse transcription loop-mediated isothermal amplification) system for Zika virus and dengue fever virus detection, where the system balanced flow rate and reaction time through delay channels, achieving detection sensitivity of 1–10 copies/reaction within 60 min [29].

Chromatographic effects utilize interaction differences between analytes and stationary and mobile phases to achieve separation, such as purifying target DNA or RNA from complex samples. However, during diffusion processes, the paper material itself may cause analyte loss due to non-specific adsorption. Ye et al. reported that analytes such as Ni^2+^ are significantly consumed during migration on cellulose substrates due to strong interactions with cellulose [35]. This phenomenon highlights the importance of selecting appropriate substrate materials. Glass fiber membranes, due to their high nucleic acid binding capacity, have demonstrated excellent performance in nucleic acid capture and concentration processes. Researchers employed UV-ozone-treated glass fiber to achieve nucleic acid purification, establishing a UVO-treated glass fiber integrated lateral flow platform with sensitivity reaching 10 CFU/mL [36]. Currently, material selection is gradually evolving from single cellulose substrates to multi-material composite systems to meet more complex detection requirements. Combining traditional cellulose matrices, such as cellulose qualitative filter paper, chromatography paper, nitrocellulose (NC) membranes, with plastic films, polymer thin films, and 3D-printed resins can significantly enhance mechanical strength, barrier properties, and chemical resistance while maintaining the low cost and capillary-driven advantages of paper materials [37]. Cui et al. designed a dual-modal paper-based microfluidic system combining colorimetric LAMP with immunochromatography [31]. The device employs wax-printed filter paper as the substrate, partitioning into colorimetric zones and ICT zones (gold nanoparticle probes). Samples are driven by capillary action through paper channels, where the ICT module enables target enrichment via digoxin-FITC dual labeling (Figure 1b). These composite structures not only exhibit excellent performance in multi-step liquid processing, long-duration reactions, and high-sensitivity nucleic acid testing but also provide material foundations for durable field detection and home-based testing.

The performance of μPADs is fundamentally governed by the synergy and antagonism between capillary-driven and multiple physical field transport processes. Among these, capillary flow rate, determined by material properties and structural design, interacts with processes such as diffusion, mixing, and chromatographic separation, collectively shaping reaction kinetics and ultimately determining detection sensitivity, speed, and reliability. Future development is shifting from simple fluid control toward precise programming of transport processes, with core strategies involving multi-material composites and structural innovations to achieve precise adaptation to different detection requirements while maintaining the low-cost and easy-operation characteristics of paper-based technology. Therefore, in μPADs design, materials, structures, and fluid parameters must be systematically optimized to balance rapid detection with sufficient reaction time, which is particularly crucial for implementing reliable multi-step reactions and multi-target analysis.

### 2.2. Patterning and Channel Fabrication Methods

In recent years, multiple automated and high-precision surface processing methods have emerged, constructing physical or chemical barriers on paper substrates to define fluid paths, achieving precise control over liquid flow direction, velocity, and distribution. This developmental trajectory has progressively enhanced the refinement level and structural complexity of paper-based microfluidic channels while expanding their functional diversity, effectively addressing early issues such as single flow modes and non-uniform distribution. This section categorizes fabrication methods into two categories for discussion: automated scalable production, including wax printing, roll-to-roll (R2R) processing, laser etching, and screen printing; and refined customization methods, including photolithography, chemical vapor deposition (CVD), and plasma treatment. Different methods exhibit distinct characteristics in terms of precision, cost, and scalability. The comparison of two major fabrication methods is available in Table 1.

#### 2.2.1. Automated and Scalable Production

The earliest technique applied to microfluidic μPADs fabrication was wax printing. This method creates hydrophobic patterns on paper substrates using wax printers, with subsequent heating causing wax penetration into fibers to form hydrophobic barriers, achieving resolution ranging from 500 μm to centimeter scale [43]. Wax printing utilizes inexpensive materials and simple processes, enabling rapid construction of fluid channels that guide liquids to reaction zones. This approach is suitable for single-step or simple multi-step reactions and can achieve multiplexed detection through multi-channel designs that distribute samples to different reaction zones [43]. However, commercial wax printers have been discontinued, prompting researchers to explore alternative solutions. For example, a fabrication technique using two-sided patterning with a thermal-transfer printer and laminator has demonstrated feasibility as an alternative to traditional wax printers [44]. This technology not only enables μPADs prototype creation without traditional wax printers but also supports roll-to-roll batch production. Its resolution is similar to or slightly higher than wax printing, achieving channel width control of approximately 100–300 μm while maintaining scalability at low cost. Simultaneously, some research has broken the binary hydrophilic/hydrophobic boundary, beginning to explore using different degrees of modification to alter contact angles and thereby change flow channel creation [45].

Cutting represents the simplest method for μPADs fabrication. Through blades, punches, or laser cutters, channels or reaction zones are physically cut on paper substrates, enabling the construction of two-dimensional or three-dimensional μPADs structures. The resolution depends on tool type, with laser cutting achieving less than 100 μm, making it suitable for rapid prototyping and small batch production [46,47]. Its advantage lies in enabling multi-channel layouts without additional chemical processing, though it faces limitations in boundary precision and complex design capabilities [48]. Zhong et al. utilized knife and laser cutting to manufacture three-dimensional μPADs, constructing multi-channel structures to achieve multiplexed pathogen detection [49]. Screen printing has recently been employed for rapid batch manufacturing of μPADs. This method uses templates to deposit hydrophobic materials such as polydimethylsiloxane (PDMS) and polyurethane, or functional inks including conductive ink and enzyme ink directly onto paper surfaces to form channels or reaction zones, exhibiting excellent repeatability and batch consistency [50]. Compared to wax printing and laser etching, screen printing demonstrates higher capacity and scalability in industrial roll-to-roll production, particularly suitable for batch manufacturing of multiplexed detection μPADs [51].

#### 2.2.2. Refined Customization Methods

In high-precision customization, photolithography represents a primary method for fabricating high-resolution μPADs. This technique applies photoresist coating on paper surfaces using photomasks and ultraviolet light, with uncured portions removed to form hydrophobic barriers, achieving resolution below 100 μm and enabling complex geometries and high-precision designs [52,53]. Photolithography enables precise fluid path control and supports integration of multiple reaction zones, thereby meeting multiplexing requirements for complex nucleic acid testing workflows [54]. However, this method typically requires several hours for fabrication cycles, and photoresist residues may interfere with subsequent biochemical reactions, resulting in higher manufacturing costs. To overcome the time-consuming and equipment-intensive nature of conventional photolithography, significant advancements have been made in rapid prototyping techniques. Martinez et al. pioneered the FLASH (Fast Lithographic Activation of Sheets) method, which enables the fabrication of paper-based microfluidic devices in under 30 min using only a UV lamp and a hotplate, without requiring a cleanroom or specialized facilities [55]. Xiong et al. designed a radial paper-based microfluidic chip. The device features a central sample inlet branching into 10 reaction-detection zone pairs, with SU-8 photoresist defining channel volumes. This approach achieves functional hydrophilic channels as narrow as 200 μm and hydrophobic barriers as small as 186 μm [56].

Plasma treatment technology, initially proposed by Martinez et al., has been used to fabricate microfluidic paper-based chips for glucose and protein measurements [16]. During plasma processing, reactive substances, ions, and electron beams generated can be manipulated through electric and magnetic fields, interacting with paper matrix surfaces to induce chemical reactions. Consequently, the formation and breaking of chemical bonds alter paper surface properties. Through plasma treatment, various effects can be achieved, including increasing paper hydrophilicity, improving wettability, enhancing surface energy and tension, and reducing capillary effects. Two common chemical formulations for rendering paper hydrophobic are PDMS and alkyl ketene dimer (AKD), with equipment for plasma treatment, including plasma cleaners and handheld corona generators. Subsequently, treated paper is placed in plasma-bonded masks for processing, with unmasked portions becoming hydrophilic while masked portions remain hydrophobic. Raj et al. utilized plasma fluorocarbon (PFE) deposition and O_2_ plasma etching to fabricate fully enclosed hydrophilic channel structures on single paper sheets, with metal masks defining channels, followed by etching to generate reagent loading holes [57].

CVD has recently been employed to directly generate hydrophobic channels within paper substrates, enabling high-precision patterning without liquid-phase chemical reaction conditions. For example, in trichlorosilane (TCS) CVD formed hydrophobic barriers on chromatography paper, thereby constructing paper-based flow channels [58]. More recently, initiated CVD (iCVD) technology has been developed. This solvent-free vapor deposition method is applicable to cellulose paper substrates [59]. By altering monomer types and deposition process parameters, iCVD can impart controllable chemical functionality to paper-based devices while accommodating roll-to-roll scale manufacturing.

Recent paper-based microfluidic patterning and channel fabrication are transitioning from laboratory prototypes to automated, scalable approaches, moving from previous methods such as wax printing, cutting, and photolithography to future high-throughput routes represented by roll-to-roll wax printing and surface modification [60], digital flexible routes represented by laser engraving and printing [61], and low-cost batch routes represented by screen printing [51]. Existing manufacturing technologies still exhibit significant trade-offs between resolution, cost, and production throughput, particularly in home-based testing scenarios where device simplicity and batch consistency are equally important. Future exploration of hybrid manufacturing strategies, supplemented by material coating optimization, could balance economic factors with detection performance. With the introduction of functional surface engineering, green materials, and flexible production lines, future μPADs manufacturing is expected to achieve unification of high precision, high consistency, and large-scale production while maintaining low cost and sustainability.

### 2.3. Structural Designs and Engineering Strategies

Over the past decade, the structural design of paper-based microfluidic chips has evolved from single-layer planar layouts to multi-layer three-dimensional integration, significantly enhancing their performance in complex biochemical reactions, fluid control, and multifunctional integration. To meet the demands of POC diagnostics for high throughput, automation, and multi-step reaction processes, researchers have developed novel configuration strategies, including 3D origami and reconfigurable designs based on traditional 1D and 2D μPADs. These designs not only optimize space utilization and functional partitioning flexibility but also enable integration of sample preprocessing, amplification, and detection through precise fluid path planning. This section will systematically introduce μPADs designs from 1D, 2D, and 3D perspectives, focusing on their functional integration, reaction efficiency, and operational characteristics.

One-dimensional μPADs (1D μPADs) rely on unidirectional capillary flow to transport samples from the inlet to the detection zone [42]. Typical structures consist of sequentially connected sample loading zones, flow channels, and reaction or detection zones, with liquid paths present in a linear fashion, often fabricated using low-cost processes such as wax printing, inkjet printing, or blade cutting [38]. These configurations offer significant advantages, including simple manufacturing, short prototype development cycles, and low cost. However, due to their fixed fluid paths and limited space utilization, 1D μPADs struggle to support multi-step chemical reactions and sample diversion, resulting in restricted controllability and integration of reaction processes. Barnes et al. developed a CRISPR-Cas13a paper-based microfluidic system featuring dual-mode readouts for enhanced flexibility [30]. The core of this diagnostic platform is a conventional lateral flow immunochromatographic test strip, adapted for nucleic acid detection. Structurally, the strip consists of sequential layers: a sample pad for initial liquid loading, a conjugate pad impregnated with streptavidin-coated gold nanoparticles, a nitrocellulose membrane with a test line and a control line, and a final absorbent pad to drive capillary flow. The innovation lies in the functionalization of the conjugate pad and test line to capture the biotin/FITC-labeled SHERLOCK amplicons (Figure 1a). In recent years, some studies have attempted to enhance functional integration by introducing time-delay zones and pre-reaction processing zones within single channels. Delay channel designs use narrower or longer channels to slow fluid flow, delaying the time for samples or reagents to reach specific regions. This ensures completion of one reaction before the next begins, preventing cross-contamination. Rodriguez et al. designed a segmented μPADs using PES membranes for nucleic acid extraction and LAMP amplification, ensuring efficient sequential processing [62]. A recent 2025 study by Siriyod et al. proposed a μPADs design incorporating wax gate structures to temporarily obstruct liquid during the amplification phase, achieving time control through regulating fluid release. This device performed recombinase polymerase amplification (RPA) and quantified *Plasmodium* nucleic acids, with a total detection time within 40 min. Additionally, 1D μPADs can be integrated with external chip structures to form functional composite systems. This fusion leverages the advantages in fluid manipulation with the analytical application strengths of paper-based platforms [63].

As multifunctional integration has become a development trend in paper-based microfluidics, their limited fluid manipulation dimensions are gradually being replaced by more powerful two-dimensional and three-dimensional structures. Two-dimensional μPADs (2D μPADs) achieve significant improvements in fluid manipulation within planar geometric space, overcoming many limitations of 1D μPADs through geometry-based flow control strategies, such as varying channel width, modifying fluid path, and integrating fluidic delays [32,56,64,65]. Fluid control in 2D μPADs primarily relies on two typical design principles: first, distributing samples from a single region to multiple different detection zones, such as Y-shaped or branched structures; second, converging multiple liquids to the same detection zone, such as petal-shaped or dumbbell-shaped configurations. To achieve high-throughput analysis in a planar format, 2D architectures often employ radial geometries to split a single sample volume into multiple detection arms. As shown in Figure 1e, this design strategy allows for the parallel quantification of distinct analytes within a single compact device (Figure 1e). These planar channel designs not only retain the intrinsic advantages of paper-based devices but also significantly enhance their functionality in multiplexed analysis [38,66]. By introducing delay channels, valve structures, and layered designs, 2D devices can achieve automatic sample distribution, timed reagent release, and sequential control of multi-step reactions. Compared to 1D μPADs, 2D designs offer advantages in space utilization, detection throughput, and parallel detection capabilities. Although 2D μPADs have achieved technological breakthroughs in multiple aspects, the precision of fluid timing control during multi-step reactions remains limited, affecting the reliability of complex analytical processes. For temperature-sensitive reactions, the lack of integrated temperature control solutions still requires connection to external equipment. Additionally, when processing complex samples, the integration degree of the preprocessing steps remains insufficient.

Three-dimensional μPADs are primarily characterized by involving liquid flow at different heights, not just liquid flow within the same horizontal plane [25]. Their core design principle is based on constructing three-dimensional fluid networks by stacking multiple layers of hydrophilic paper-based materials to achieve complex biochemical analysis functions [67,68]. To simplify the workflow for end-users, origami-inspired architectures have been developed to integrate multi-step protocols. For instance, Sun et al. designed a foldable paper device that combines on-chip cell lysis with colorimetric signal amplification, allowing for the sensitive detection of *E. coli* without external pumping systems (Figure 1g) [69]. Toldra et al. reported a microfluidic enzyme-linked paper analytical system using multilayer structures to implement enzyme-linked detection processes: capable of performing elution and color development steps and initiating reactions through folding; after RPA, it achieved an LOD and LOQ as low as <10 genome copies/μL; performance improved by at least 70-fold and 1000-fold compared to traditional gold nanoparticle lateral flow assay (LFA); this device is simple to manufacture while maintaining high-sensitivity quantitative analysis capabilities [34]. Additionally, multilayer μPADs technology effectively addresses the limitations of traditional paper-based devices with slow flow rates and inability to perform complex field analysis. Three-dimensional μPADs introducing vertical gaps > 300 μm achieve significant flow efficiency enhancement, with flow rate increases up to 169-fold. Such structures break the predictive model of capillary flow in the traditional Lucas-Washburn equation. Theoretically, this dramatic acceleration is attributed to the elimination of the cellulose fiber network within the hollow channels. This structural modification removes the high hydraulic resistance inherent to porous media, shifting the transport mechanism from Darcy-type wicking to open-channel capillary flow, where velocity is governed by channel geometry rather than pore permeability [70,71]. Xu et al. developed origami-style μPADs that achieved nucleic acid extraction and amplification through folding, with DNA recovery rates of 60%. Each layer can be designed as a specific functional zone, with multi-step reaction integration achieved through folding alignment [72]. Rohrman et al. utilized origami-style μPADs to integrate sample lysis and later flow detection, shortening detection time in 15 min [73]. Through multi-channel or porous designs, samples are distributed to different reaction zones, supporting multiplexed detection.

However, nucleic acid diagnostics using μPADs still face several critical bottlenecks. The inherent non-uniformity and background interference of paper-based materials may affect detection sensitivity, particularly in cases of low target concentration. When performing nucleic acid extraction and purification on paper-based substrates, sample loss and inhibitor residues may still lead to poor detection limits, making stable detection and precise quantification at low target concentrations difficult to achieve [74,75]. Ideal POC devices should achieve “sample-in, result-out” functionality. However, efficiently integrating multiple steps, including nucleic acid extraction, purification, amplification, and detection into a single paper-based device, while ensuring seamless connections between steps and avoiding contamination, remains a significant challenge. Although some studies have attempted to integrate blood preprocessing or utilize 3D paper structures for integrated sample preprocessing, amplification, and detection, how to automate and miniaturizing these steps while ensuring their stability and reliability still requires in-depth research [72,76]. Additionally, the precision and flexibility of fluid control need improvement. Currently, many paper-based nucleic acid testing technologies remain at the laboratory proof-of-concept stage, with relatively limited performance, stability, and large-scale clinical validation in complex matrices such as whole blood, sputum, and saliva [77].

Modern μPADs demonstrate significant advantages in simplicity, cost-effectiveness, stability, storability, disposability, and portability [68]. These technological advances enable multilayer μPADs to have broad application prospects in pathogen nucleic acid detection and food safety, representing an important trend in the development of microfluidic technology toward low-cost, high-performance directions.

## 3. Nucleic Acid Testing Process Based on Amplification

Compared to traditional laboratory testing, the core challenge of home-based nucleic acid testing lies in achieving seamless integration of the entire workflow from nucleic acid extraction, nucleic acid amplification, to signal detection on a single paper-based device [78,79]. Multi-layer paper-based microfluidic architectures proposed in recent years functionally integrate sample processing layers, reaction amplification layers, and detection readout layers. Through optimization of capillary-driven microchannels, reagent carriers that can be stored at room temperature, and low-temperature isothermal amplification technologies such as RPA and LAMP, μPADs have made significant progress in portability and user-friendliness, with the potential to achieve a true ‘sample-in-result-out’ closed loop [78,80]. Additionally, combining smartphones or low-cost optical modules for signal capture and quantification can further reduce user operation errors, enable automatic interpretation and remote data sharing, providing scalable digital solutions for home testing [81,82]. From an interdisciplinary perspective, the future development of μPADs depends not only on innovations in molecular biology and materials chemistry but also requires collaborative optimization of microfluidic engineering, nano-functional materials, and data science to meet multidimensional needs in areas such as home use, food safety, and public health screening. Overall, as home-based nucleic acid testing platforms, the technological maturity and interdisciplinary integration capabilities of μPADs will determine their application potential in responding to future infectious disease outbreaks and routine health management.

To overcome these barriers, it is essential to deconstruct the μPADs workflow into its critical functional modules. The reliability of a home-based diagnostic system relies heavily on the seamless coordination of upstream nucleic acid extraction, isothermal amplification, and downstream signal readout. The following sections critically review the technological innovations and integration strategies within these three dimensions, evaluating how each contributes to the realization of robust, sample-to-answer platforms

### 3.1. Nucleic Acid Extraction and Enrichment: Sample to Template

Sample preprocessing represents a critical starting point for μPADs in molecular diagnostic applications, with the core task of efficiently separating and concentrating DNA or RNA from complex samples such as blood, urine, saliva, and nasal swabs, thereby providing stable and high-quality templates for subsequent amplification and detection [83,84,85]. Traditional methods rely on centrifugation, filtration, or expensive equipment, whereas μPADs integrate key steps including lysis, capture and enrichment, and purification on a single platform, enabling low-cost, instrument-independent nucleic acid processing.

**Figure 2:**
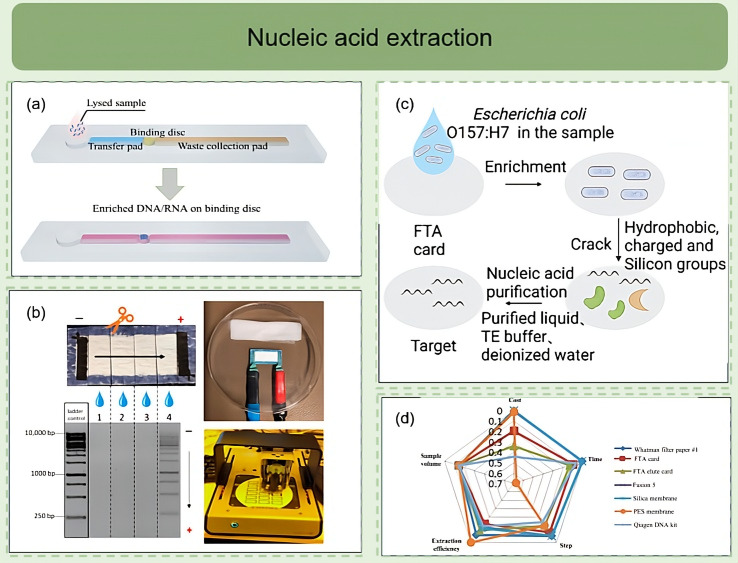
Schematic comparison of four distinct paper-based NA extraction and purification methodologies. (**a**) A lysed sample is passed through the paper matrix, where NA is physically adsorbed onto the cellulose fibers. Subsequent washing with a buffer removes inhibitors, and NA is finally recovered with a small volume of elution buffer. The entire process is passive and equipment-free [86]. (**b**) Upon application of an external electric field across the µPADs, negatively charged DNA molecules migrate electrophoretically, the number represented as 1, 2, 3, 4 was the different part of the fiber chip [35]. (**c**) FTA card-based integrated extraction for direct detection. This approach consolidates cell lysis and NA immobilization into a single step [87]. (**d**) Systematic comparison of paper-based nucleic acid extraction materials. This work provides a benchmarking framework rather than a novel method [91].

The lysis phase typically disrupts cellular structures through chemical or physical means to release nucleic acids. Chemical methods such as SDS, Triton X-100, or alkaline lysis solutions can rapidly dissolve cell membranes within paper-based channels [86]. As illustrated in Figure 2a, integrating lysis and enrichment zones in multilayer paper channels enables continuous solution transfer through interlayer capillary forces, completing nucleic acid extraction within 30 s. Capture and enrichment depend on material selection and chemical modification. Common strategies include fixing cationic groups on filter paper or FTA cards to capture DNA/RNA molecules through charge adsorption or hydrogen bonding [86]. The FTA card integrated system constructed by Chen et al. achieved rapid extraction of *E. coli* O157:H7 from stool samples, with a sensitivity approximately 100-fold higher than traditional methods [87] (Figure 2c). Wang et al. reported a continuous-flow microfluidic extractor using magnetic silica beads to capture and purify DNA from bacterial samples, achieving a lower detection limit of ~10^2^ CFU·mL^−1^ for *Salmonella* when coupled with on-chip qPCR; Suaifan and Hao et al. untilized the paper- or chip-based approaches that use magnetic nanoparticles for enrichment have reported limits of detection in the 10^1^–10^2^ CFU·mL^−1^ range for *E. coli* and *Salmonella*, supporting the feasibility of magnetic-enrichment strategies for clinical and food-safety testing [88,89,90].

The purification phase implements automated washing and separation through hydrophilic and hydrophobic interface design. Contact angle differences between channels promote sequential solution flow through washing and collection zones, avoiding manual intervention and reducing sample loss. Another representative approach involves electrophoretic-driven μPADs extraction systems, which utilize electric field migration to achieve DNA purification and graded enrichment without external pumps [35] (Figure 1f). The anode and cathode regions connect through conductive paper fiber layers, enabling nucleic acid migration to target zones while removing impurity proteins. Addressing performance differences among various paper-based materials, Tang et al. systematically compared nucleic acid binding efficiency and elution recovery rates of materials such as Whatman Grade 1, FTA, FTA elute card Fusion 5, Silica membrane and Polyethersulfone (PES) membrane. Results showed that FTA cards and glass fiber paper achieved approximately 80% nucleic acid recovery rate in blood and saliva, while nitrocellulose membranes were more suitable for capturing low template concentration samples [91] (Figure 2d). μPADs utilize paper-based materials, particularly glass fiber paper, for simultaneous lysis and purification due to their high nucleic acid binding capacity, biocompatibility, and impurity filtration capabilities [92]. However, these materials exhibit sensitivity to operating conditions regarding wet strength requirements, necessitating strict control of pore uniformity during preparation to avoid batch-to-batch variations. The extraction methods are summarized in Table 2.

μPADs demonstrate unique comprehensive advantages in sample preprocessing, although the technology still faces challenges in material stability, precision, and scalability. The insufficient durability of paper-based materials in high humidity or extreme pH environments, along with stabilization production challenges posed by nanomaterials and complex configurations, present significant hurdles. However, recent original studies have demonstrated that targeted material optimization and packaging strategies can substantially improve μPADs performance under high-humidity and field conditions. For instance, the development of acid-free paper-based analytical devices by Rawat et al. [95] showed enhanced stability in high-humidity environments, while the creation of superhydrophobic eggshell coatings by Thangjitsirisin et al. [96] provided excellent moisture resistance for field applications. These material advancements make μPADs more suitable for preprocessing of non-invasive samples such as sweat, tears, and saliva, which often require collection in challenging environmental conditions [41,97]. Future prospects involve combining portable printing, printable hydrophobic materials, bio-based cellulose, and other sustainable solutions, while introducing artificial intelligence-assisted design and modular architectures. These approaches are expected to further achieve low-cost, high-precision, customizable sample preprocessing. In recent years, application sample types have expanded from blood and urine to non-invasive body fluids such as respiratory condensate, tears, and sweat, providing novel technological pathways for rapid field detection and continuous health monitoring [98].

### 3.2. Nucleic Acid Amplification: Enhancing Speed and Portability

μPADs combined with isothermal nucleic acid amplification technologies have emerged as highly promising POC platforms for infectious disease diagnosis. Unlike traditional PCR methods requiring complex thermal cycling equipment, isothermal amplification techniques—including LAMP, RPA, and nucleic acid sequence-based amplification (NASBA)—operate under constant temperature conditions, making them ideal choices for μPADs applications. LAMP employs Bst polymerase with strand displacement activity at 60–65 °C, while RPA utilizes recombinases to facilitate primer binding at 37–42 °C, with reaction times of 10–20 min and enhanced tolerance to crude samples. The low voltage requirements for isothermal amplification enable temperature control through various portable methods, including printed circuit board (PCB) heaters, chemical heaters, or body heat utilization.

Multiple isothermal amplification μPADs detection platforms have been developed in recent years. Mei et al. developed a portable μPAD capable of extracting SARS-CoV-2 and Helicobacter pylori nucleic acids from saliva or swab samples, with LODs of 4 × 10^2^ copies/mL and 1 × 10^3^ counts/mL, respectively, and a total detection time of less than 30 min [86]. This system utilizes FTA cards for nucleic acid enrichment combined with RPA, demonstrating excellent field deployment capabilities. For malaria detection in resource-limited regions, Reboud et al. developed a μPADs using LAMP at 60–65 °C to detect *Plasmodium*, integrating lateral flow analysis and magnetic bead DNA concentration technology, achieving an LOD of 32 copies/reaction within 50 min [99]. Seok et al. presented μPADs performing LAMP at 63 °C for real-time detection of multiple DNA targets, using hydroxy naphthol blue fluorescence to achieve a detection range of 10^2^–10^5^ copies of genomic DNA within 60 min [100]. Rohrman and Richards-Kortum developed paper and plastic devices for HIV DNA RPA at 42 °C to detect the background DNA tolerance, using lyophilized enzymes and lateral flow strips to amplify 10 copies of HIV DNA to detectable levels within 15 min [73]. To provide a comprehensive overview of the current landscape, Table 3 summarizes representative studies on nucleic acid detection using PADs. This comparison highlights key performance metrics, including detection limits, assay time, and device cost, across different amplification strategies.

Simultaneously, the integration of emerging CRISPR technologies with μPADs provides new opportunities for achieving ultra-sensitive and highly specific nucleic acid detection. Lesinski et al. constructed a paper-based microfluidic device integrating RPA and CRISPR-Cas12a [114]. This system achieves full-process integration of sample input, isothermal amplification, and Cas12a signal amplification by embedding a dual-channel valve-controlled fluidic network in the μPADs. Utilizing linear and circular reporter molecule designs, the researchers achieved an LOD improvement from 1 pM to 1 fM, with detection time shortened to 35 min, demonstrating the feasibility of CRISPR for precise virus screening on paper-based platforms. Another study by Pan et al. [115] demonstrated a CRISPR/Cas12a-driven SERS biosensor for ultrasensitive nucleic acid detection. This system tactfully combined the specific recognition of CRISPR/Cas12a with a Prussian blue nanolabel-modified probe, achieving signal amplification through the release of PB nanoparticles upon target DNA recognition. The method exhibited an exceptionally low LOD of 224 aM for target DNA and was successfully applied to detect cow milk adulteration in goat milk. Although this design relies on a portable Raman spectrometer for readout, its fusion of nano-optics with molecular recognition provides a new technical approach for high-sensitivity on-site detection in food matrices [115].

Despite these advances, the widespread application of μPADs technology still faces standardization challenges. Ensuring consistent detection performance across different production batches and environmental conditions requires establishing unified quality control protocols. Temperature control precision, particularly in home-use scenarios, constitutes a technical bottleneck that directly impacts detection reliability. Additionally, enzyme storage stability presents logistical challenges, as maintaining enzyme activity while simplifying cold chain requirements remains unresolved

### 3.3. Signal Detection and Data Processing: From Qualitative to Quantitative

The effectiveness of μPADs critically depends on converting biochemical reactions on paper substrates into measurable signals for qualitative, semi-quantitative, or quantitative analysis. With the integration of information processing technology, mobile applications, and the Internet of Things (IoT), μPADs have demonstrated significant enhancements in automated analysis, real-time monitoring, and remote data transmission, further improving diagnostic efficiency and accessibility.

Among signal detection methods, colorimetric detection represents the most widely applied technology. This approach relies on color changes induced by enzymatic reactions, pH indicators, or redox reactions, establishing correlations between color intensity and analyte concentration. Fluorescence detection provides enhanced sensitivity for low-concentration analytes such as nucleic acids or low-abundance biomarkers. Mei et al. combined fluorescence detection with RPA technology, achieving SARS-CoV-2 RNA detection on μPADs with a LOD of 4 × 10^2^ copies/mL within 30 min [86]. Electrochemical detection offers high sensitivity and excellent compatibility with portable devices by measuring current, voltage, or impedance changes from electrochemical reactions on paper-based electrodes. Wang et al. further developed a μPADs system utilizing magnetic nanoparticles for DNA extraction from pathogenic bacteria, achieving differential detection of redox peak currents through charge changes caused by DNA probe immobilization and hybridization reactions on working electrode surfaces [116]. Chemiluminescence and electrochemiluminescence detection methods maintain high sensitivity without external light sources, making them suitable for detecting analytes such as miRNA that cannot be amplified [117,118]. Yang et al. reported an ECL sensor for miR-21 with a linear range from 0.01 pM to ~10 nM [119]; carbon-dot ECL systems have achieved 10 fM detection [120]. Additionally, labeling technologies based on gold or silver nanoparticles effectively enhance signal intensity and support multiplexed detection. Xie et al. employed gold nanoparticle-enhanced CRISPR detection systems in μPADs, achieving an LOD of 1 fM [121].

In quantitative analysis, smartphone applications and portable readers enable precise conversion of signal intensity to concentration values. Software such as ColorScan can automatically quantify colorimetric signals in μPADs images with accuracy comparable to manual methods [122]. Quan et al. utilized sA ratiometric fluorescent nanofiber sensor achieving an LOD of 85 ppmin rBiogenic aminesdetection [123]. Dong et al. developed portable RT-LAMP μPADs equipped with intelligent image acquisition systems, requiring only 2.2 μL of reagents per test [124]. Furthermore, the deep integration of μPADs with digital technologies is driving functional innovation. Fiedoruk et al. applied machine learning algorithms to analyze fluorescence patterns from CRISPR-based detection, significantly improving pathogen identification specificity while reducing false-positive rates [125]. Cao et al. integrated RT-LAMP with CRISPR-Cas12a in paper-based devices, enabling SARS-CoV-2 detection in wastewater within 1 h [101].

The integration of multiple signal detection methods with digital technologies in μPADs establishes a solid foundation for next-generation point-of-care diagnostics. Rapid response times of 10–60 min support timely decision-making in community or home environments, while IoT applications enable real-time data sharing, facilitating disease tracking and optimized resource allocation by health authorities. As technologies continue to advance in standardization and environmental stability, μPADs will play increasingly important roles in global health, food safety, and environmental monitoring, making significant contributions to improving healthcare accessibility and health outcomes in resource-limited regions.

## 4. At-Home Point-of-Care Applications

Medical diagnosis is undergoing a transformation from centralized laboratories to home settings, a shift that has become important in the context of population aging, frequent emerging infectious diseases, and increasing patient pursuit of autonomy. The scope of home health monitoring now extends far beyond traditional measurements of body temperature and blood pressure, gradually encompassing molecular-level detection of pathogens, reproductive health markers, and specific biomarkers applications previously limited to clinical laboratories due to technical complexity and equipment requirements. μPADs have emerged as a highly promising platform. This section explores four key application areas of μPADs in home health management, collectively reflecting the technology’s significant potential in addressing current diagnostic bottlenecks. These include: epidemic screening, whose necessity was highlighted following the COVID-19 pandemic; foodborne pathogen detection addressing severe public health challenges; reproductive health screening that effectively overcomes privacy, stigma, and accessibility barriers; and disease biomarker monitoring requiring technological breakthroughs to achieve precision health management. The following text evaluates recent technological advances of μPADs in these fields, examining their analytical performance and clinical validation evidence, aiming to clarify the promotions and challenges for transitioning from laboratory prototypes to mature applications.

### 4.1. Home-Based Screening for Respiratory Epidemics

Respiratory infectious diseases are primarily caused by influenza viruses, SARS-CoV-2, respiratory syncytial virus (RSV), rhinoviruses, adenoviruses, parainfluenza viruses, and human metapneumovirus [126,127]. These pathogens frequently present with similar clinical symptoms, making differential diagnosis based solely on symptomatology challenging. The design of μPADs platforms for respiratory pathogen detection typically addresses three critical considerations. First, given the complex etiological spectrum of respiratory infections, clinical differential diagnosis necessitates simultaneous detection of multiple pathogens [128], rendering multiplexed detection platforms particularly crucial. Second, paper-based devices face an inherent trade-off between minimal sample volume requirements and high analytical sensitivity; consequently, signal amplification strategies become pivotal for reliable readout [129]. Third, field deployment presents multiple operational challenges, including reagent stability at ambient temperature and variability introduced by user self-sampling [129].

To address multiplex pathogen detection, Dinh Vu Phong et al. developed a paper microdevice integrating DNA extraction, LAMP, and colorimetric detection for respiratory pathogens including SARS-CoV-2 [130]. The device features a simplified architecture comprising a sample chamber and reaction chamber, utilizing a sealing membrane as the base layer to create a foldable structure that facilitates straightforward operation suitable for point-of-care settings. Through colorimetric readout, this device delivers results within a short timeframe, demonstrating the practicality and cost-effectiveness of paper-based microfluidics for rapid respiratory virus detection [130]. In another study, researchers developed a multiple-aptamer recognition platform MARQ-LFIA, that combines three different aptamers targeting multiple sites of the SARS-CoV-2 N protein with quantum dot colorimetric and fluorescence enhancement to construct a highly sensitive lateral flow immunoassay strip. This platform achieved a LOD of 1.427 pg/mL for N protein and 1643 U/mL for inactivated SARS-CoV-2, with a detection time of approximately 15 min. The system demonstrated 86.67% clinical sensitivity when validated with 30 COVID-19 positive clinical samples [131].

Paper-based microfluidic devices may encounter sensitivity limitations when analyzing respiratory specimens, particularly in scenarios involving low pathogen concentrations. To overcome these constraints, recent investigations have employed airflow-based evaporative methods to achieve substantial signal amplification. Edward Wang and colleagues demonstrated an airflow-based, evaporative method capable of manipulating fluid flows within paper membranes to offer new functionalities for multistep reagent delivery and improve the sensitivity of μPADs by 100–1000-fold [132]. For colorimetric LAMP detection of the COVID-19 genome, solution enrichment on paper enhances dye contrast to more clearly distinguish between positive and negative results, achieving a sensitivity of 3 copies of SARS-CoV-2 RNA. Furthermore, integration with electrochemical biosensors, colorimetric detection, or fluorescence-based methods can significantly enhance signal output sensitivity, with paper-based electrochemical biosensors emerging as a particularly active research direction in recent years [133].

A critical challenge in field-based testing lies in simplifying sample processing and reaction workflows to minimize user-induced errors. To address this issue, Liu et al. designed an integrated microfluidic nucleic acid detection chip that incorporates RT-RPA and lateral flow readout within a sealed system, achieving a complete sample-to-answer workflow in approximately 30 min with a detection limit of 1 copy/μL and demonstrating 97% sensitivity and 100% specificity in clinical specimens [134]. More recently, Wu et al. reported a 3D μPADs integrated with lateral flow-LAMP, which combines 3D paper architecture with enclosed lateral flow strips in a LAMP amplification system [135]. The device incorporates pre-dried reagents and air/aerosol isolation design to enhance amplification efficiency while reducing operational errors, achieving a detection limit as low as approximately 10 fg per reaction in a validation cohort of 815 patient samples [135].

**Figure 3:**
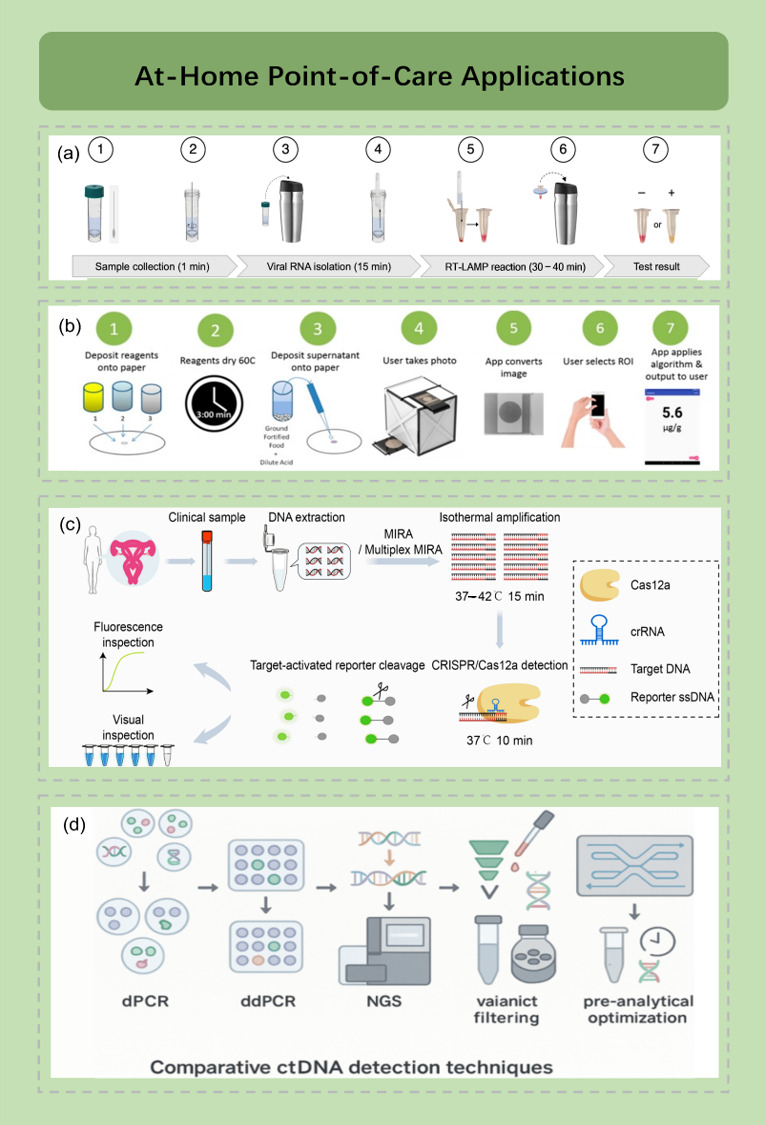
The application of at-home point-of-care nucleic acid testing. (**a**) Schematic illustration of the COVID-19 home test from sample collection to test result readout. ① sample self-collection using a nasal swap; ② swap elution in collection buffer; ③ heat treatment for sample lysis; ④ cooling and sample transfer; ⑤ adding sample to lyophilized RT-LAMP reagents; ⑥ isothermal amplification at 65 ℃; ⑦ visual colormetric readout of results [136]. (**b**) A paper-based colorimetric assay for rapid at-home food safety screening. Schematic for the procedure for preparing the paper-based assay, sample deposition, and analysis the reagents in step 1 were Reagent 1, hydroxylamine hydrochloride. Reagent 2, ferrozine. Reagent 3, ammonium acetate buffer. ① Deposit reagents onto paper; ② waiting for dry; ③ deposit supernatant onto paper; ④ user takes photo; ⑤ app converts images; ⑥ user selects ROI; ⑦ app applies algorithm and out put results [139]. (**c**) Illustration of the CRISPR/Cas12a-based fluorescent assay for HPV detection in clinical samples [140]. (**d**) An overview of representative analytical techniques for circulating tumor DNA (ctDNA) detection, including dPCR, ddPCR, and NGS-based approaches [141].

Nucleic acid testing in resource-limited settings is constrained by cold-chain requirements and equipment dependencies. Addressing the need for home-based testing, Song et al. developed a lyophilized RT-LAMP colorimetric detection kit that enables sample lysis, amplification, and visual color-change interpretation without sophisticated equipment. The reagents maintain stability at room temperature for six weeks, demonstrating promising potential for field deployment [136] (Figure 3a). A complementary study systematically evaluated the impact of different lyoprotectant formulations on the thermal stability of LAMP systems, revealing that activity can be maintained for over one month at 37 °C [137]. For user-friendly deployment, result interpretation in field or home diagnostic settings should leverage portable readout modalities, with smartphones representing an optimal output terminal. Sen et al. developed the paper-based PLACID platform, which couples LAMP with CRISPR/Cas12a on a paper chip. Through reagent pre-drying combined with infrared heating and smartphone-based fluorescence imaging, the system reports a detection limit of approximately 50 copies/μL for SARS-CoV-2 RNA fragments [138].

### 4.2. Rapid At-Home Screening for Food Safety

Foodborne disease outbreaks represent a persistent threat to global public health. According to global estimates, contaminated food causes approximately 600 million illnesses and 420,000 deaths annually [142]. Studies indicate that in developed countries, a significant proportion of foodborne disease cases, estimated at 30–40%, are associated with improper home food handling [143,144]. μPADs offer a viable pathway for household-level food safety monitoring due to their rapid response, low cost, and requirement for minimal specialized equipment. Key targets for food safety detection include major bacterial and viral pathogens. The primary challenges for nucleic acid testing in home food safety applications center on sample complexity, detection stability, and balancing sensitivity with specificity. Food samples contain lipids, proteins, polysaccharides, and small-molecule inhibitors that can directly inhibit enzymatic amplification or mask signals in paper-based detection workflows, ultimately causing false negatives or reduced sensitivity. Consequently, inhibitor removal or target enrichment must be incorporated as core modules in these systems [145].

Matrix inhibition effects on amplification and detection are commonly addressed through strategies such as extraction and purification, sample dilution, additive incorporation, or employing inhibition-resistant polymerases. However, these approaches involve trade-offs: dilution reduces inhibition while sacrificing target nucleic acid concentration, and chemical inhibitor removal steps increase operational complexity [146]. To address the challenge of high recovery and rapid extraction in instrument-free environments, paper-based strategies provide an effective pretreatment solution due to their inherent filtration properties. Chen et al. developed a portable origami microfluidic device that enabled rapid detection of *Salmonella enterica*, incorporating nucleic acid extraction on paper dipsticks without pipetting, nucleic acid amplification, and lateral flow assay for result readout [147]. This study utilized polyethersulfone membrane as a novel reaction matrix instead of widely used chromatography paper to optimize nucleic acid amplification, achieving a sensitivity of 260 CFU/mL within 20 min, equivalent to RPA reactions in tubes. Alternatively, some research focuses on optimizing amplification systems to mitigate the effects of inhibitory components in samples. Moon et al. summarized evidence of polysaccharide and polyphenol inhibition in LAMP/RPA from fruits and vegetables, listing case studies where amplification activity was restored through surface modification or localized chemical neutralization [148]. For home or field testing, solutions that integrate efficient inhibitor removal with high recovery rates in a single step remain lacking. Therefore, systematic evaluation of different inhibitor removal methods for nucleic acid recovery rates in typical food matrices is necessary to determine which strategies most effectively enhance detection sensitivity.

Through integration of advanced sensing technologies, paper-based microfluidic devices show promise for achieving high-sensitivity, multiplexed detection in home and field settings. Sandeep B Somvanshi et al. reported a novel paper-based single-input channel microfluidic device capable of simultaneously detecting multiple whole-cell foodborne bacteria, with quantitative reading enabled through image analysis [149]. This device utilizes multiplexed aptasensors to simultaneously detect *E. coli* O157:H7 and *S. typhimurium*, avoiding the complexity of traditional devices requiring multiple independent channels and enhancing multiplexed pathogen screening capabilities. Similarly, multiplexed colorimetric paper-based microfluidic devices can detect and differentiate multiple target bacteria, suitable for rapid screening of water, food, and agricultural products [149]. Combined with smartphone applications, paper-based microfluidic devices enable rapid, convenient data reading. For instance, applications can detect color changes in paper-based devices through RGB analysis, directly identifying multiple foodborne pathogens and providing both visual and quantitative outputs. This approach reduces the need for specialized equipment, with LOD as low as approximately 10 CFU/mL, demonstrating superior sensitivity compared to traditional enzyme-based methods [150]. Another MDC@N-MMCNs paper-based sensor, paired with a smartphone application, achieved selective detection of multiple foodborne pathogens with a visual LOD of 10 CFU/mL, supporting rapid result acquisition in field environments [151].

The high specificity of CRISPR/Cas systems effectively enhances the reliability of detection results. Lee and Oh reported a *Salmonella* detection system combining LAMP with CRISPR/Cas12a in tandem, visualized through lateral flow strips. Their approach employed sample filtration or simplified pretreatment to remove large particles and some inhibitors, followed by LAMP amplification in a closed reaction chamber to trigger Cas12a trans-cleavage activity, with final visual interpretation achieved through improved lateral flow strip band intensity reading. This work demonstrated high specificity in food matrices including onions, melons, and cured meats, with significantly lower false-positive rates compared to traditional LAMP, and reported detection limits approaching 10^1^–10^2^ CFU/g. This method seamlessly couples CRISPR’s high specificity with lateral flow visualization, reducing instrument dependency while enhancing field readability [152]. Gong et al. reported a one-pot LAMP-CRISPR platform for *Salmonella typhimurium* that accomplished both amplification and CRISPR identification in a single tube. By optimizing enzyme ratios, timing sequences, and buffer systems, this approach enabled reliable triggering of LAMP amplification with subsequent or parallel Cas12b-mediated cleavage in the same closed reaction system, thereby avoiding contamination from open transfers and reducing total time to 30–45 min [153]. The reported detection limit approached tens of copies, with trace detection validation in meat samples. The innovation lies in engineered “reaction compatibility” optimization, facilitating deployment in field and semi-automated scenarios [153]. For instance, a mobile phone-assisted paper sensor was reported for the rapid, on-site screening of iron formulas in fortified flours without requiring bulky instrumentation (Figure 1b) [139].

Paper-based microfluidic technology plays a critical role in foodborne pathogen detection, with core applications including field multiplexed detection, smartphone-assisted analysis, and integration of aptamers and CRISPR systems, enabling portable, low-cost, and sensitive screening [149,150]. These devices demonstrate significant potential in food safety applications but require addressing interference and manufacturing challenges to enhance practicality and accuracy [154].

### 4.3. Discreet and Private At-Home Screening for Reproductive Health

Nucleic acid testing for reproductive health faces significant screening coverage limitations in traditional healthcare systems due to privacy concerns, social stigma, and geographical barriers. According to 2022 World Health Organization data, cervical cancer screening coverage is only 19% in low-income countries and 33% in middle-income countries, with major obstacles including lack of trained clinicians, patient resistance to pelvic examinations, and cultural taboos [155,156]. Sexually transmitted infection (STI) screening faces even more severe social stigma challenges, with approximately 60% of adolescents refusing clinic-based testing due to privacy concerns [157,158]. μPADs are rapidly advancing by leveraging their privacy advantages through self-sampling and at-home testing models. Unlike rapid infectious disease testing, reproductive health screening emphasizes multiplexed typing capabilities, extremely low false-positive rates, quantitative analysis, and user-friendly result interpretation (Figure 3c) [140,159,160]. These specific requirements have driven innovations in μPADs technology for multiplexing, signal amplification, and readout strategies.

Different human papillomavirus (HPV) subtypes exhibit varying carcinogenic risks, making multiplexed subtyping clinically crucial. Currently, CRISPR-based technologies provide a feasible pathway for this application. For instance, Guan et al. systematically evaluated the mismatch tolerance of CRISPR-Cas12a and engineered crRNAs with specific mismatches, successfully endowing them with dual functions of approximate matching and precise querying, enabling simultaneous detection of related sequence groups or specific single subtypes [161]. Building on this approach, Guan et al. utilized spatial partitioning technology on paper-based chips to integrate different crRNAs, achieving multiplexed typing of 14 high-risk HPV subtypes [161].

For primary screening scenarios requiring high sensitivity, researchers have developed comprehensive workflows integrating sample preparation, isothermal amplification, and CRISPR and colorimetric readout on paper-based platforms to enable rapid interpretation after self-sampling at home or community healthcare points. Liu et al. innovatively integrated RPA with CRISPR-Cas12a technology on a three-layer foldable paper-based chip [162]. This system was demonstrated in clinical validation: testing 50 cervical swab samples revealed sensitivity and specificity of 95% and 100%, respectively, for the HPV16 E7 gene, with performance highly consistent with laboratory gold-standard quantitative PCR results [162].

The demand for quantitative reading in home testing is increasing, particularly for populations requiring long-term antiviral treatment. A related study reported a CRISPR-assisted nucleic acid quantification method integrated into a μPADs with signal readout by a personal glucose meter (PGM). By retaining magnetic beads on filter paper and lyophilizing all necessary reagents with trehalose stabilization, the system enables indirect quantification of HPV DNA through PGM readout without complex user intervention or reagent handling. The calculated limit of detection was 57 pM, comparable to other CRISPR-based nucleic acid detection methods. This fully integrated device demonstrated excellent storage stability for up to 4 weeks, indicating its suitability for practical point-of-care nucleic acid quantification [163].

Microfluidic paper-based analytical devices demonstrate significant potential in reproductive health testing, yet their development path reveals a tension between performance and accessibility. While integrated “sample-to-answer” workflows simplify operation, their extreme dependence on user self-sampling quality constitutes a critical vulnerability, as any deviation in sample collection can lead to erroneous results. Multiplexed typing enabled by CRISPR programmability can distinguish similar sequences but faces challenges with narrow dynamic ranges, where signals from high-abundance targets in mixed infections can easily mask low-abundance subtypes, resulting in underreporting. Simultaneously, strategies pursuing ultimate sensitivity may amplify background noise, increasing false-positive risks. High-throughput platforms relying on precise fluid control and imaging readout sacrifice the simplicity and low cost necessary for home settings, remaining dependent on community hospitals rather than representing truly decentralized solutions. Therefore, future breakthroughs will likely emerge not from linear optimization of single technologies but from balancing these interdepaendent factors.

### 4.4. Screening for Emerging Biomarkers

With the advancement of precision medicine, circulating nucleic acid biomarkers such as microRNA (miRNA), circulating tumor DNA (ctDNA), and exosomal RNA have demonstrated increasing value in early disease screening and treatment monitoring. These biomarkers can provide disease signals up to 48 months before imaging or clinical symptoms appear [164]. However, unlike the previously discussed applications, circulating biomarker detection faces numerous challenges: serum miRNA concentrations are at the femtomolar to picomolar range, ctDNA comprises only 0.01–10% of total cell-free DNA, and high RNase activity in blood can degrade 50% of miRNA within 30 min at room temperature (Figure 3d) [141]. Additionally, distinguishing physiological fluctuations from pathological changes is necessary. These characteristics make circulating biomarkers the “final frontier” for expanding μPADs technology to home applications and represent a current research hotspot.

Integrating efficient enrichment modules with paper-based detection modules offers a viable solution. Paper-based electrochemical biosensors utilizing AuNPs/RGO or AuNPs/MoS_2_ modified electrodes enable rapid detection of miRNA-21 and miRNA-155, though their detection limits remain at the nanomolar level, demonstrating the feasibility of paper-based platforms for low-abundance nucleic acid detection [165]. Furthermore, to achieve lower detection limits, researchers have introduced spatial amplification and multi-level signal amplification mechanisms in paper-based or microfluidic platforms, combined with nanomaterials to enhance transduction signals, enabling detectable signals even with minimal sample input [166]. Consequently, when applying μPADs to detect circulating nucleic acid biomarkers in home or field settings, enrichment and signal amplification strategies must be employed to overcome detection disadvantages caused by extremely low biomarker abundance.

Even when technology can detect low-abundance circulating biomarkers, translating detection results into usable clinical or home judgments remains a bottleneck. Since biomarker levels are influenced by multiple factors including physiological activities, metabolic states, and inflammatory responses, the positive predictive value of a single test may be very low. In low-prevalence populations, even with 95% specificity, the positive predictive value may be only a few percentage points. To address this situation, μPADs research has proposed multi-marker joint detection strategies, which involve detecting multiple miRNAs, ctDNA mutations, or exosomal markers on the same paper-based platform to enhance discriminative capability. A paper-based dual-channel electrochemical approach for detecting miRNA-155 and miRNA-21 demonstrated superior performance in multi-target recognition compared to single-target detection [167].

Despite these emerging applications showing broad prospects, their transition to home settings still faces numerous challenges. standardization issues persist, as batch-to-batch variations in paper substrates and environmental temperature and humidity changes can affect detection performance, necessitating the establishment of strict quality control systems. Finally, regulatory and ethical issues present significant hurdles, particularly for high-risk applications such as cancer screening, where false negatives may delay treatment and false positives may lead to overtreatment. Establishing effective linkage mechanisms between home-based testing and clinical confirmation is crucial for technology promotion. Future development should focus on integrating full-process automation, multiple internal reference validation, and cloud-based data management systems to achieve truly reliable and accessible home molecular diagnostics.

## 5. Challenges and Perspectives of At-Home Point-of-Care μPADs

Despite significant technological advances in μPADs, critical challenges remain that limit their widespread clinical application and commercialization. These challenges span manufacturing consistency, signal detection reliability, analytical performance, and system integration, requiring systematic approaches to transition from laboratory prototypes to manufacturable products.

The scalability of μPADs is fundamentally constrained by variations in channel geometry, surface energy distribution, and chemical pretreatment uniformity across batches. Inconsistent capillary flow rates between batches directly amplify reaction kinetics variations and readout instability, constituting a critical manufacturing bottleneck. As emphasized in the critical review by Gong and Sinton [14], advancing μPADs from academic prototypes to broad diagnostic applications requires rigorous control over fabrication tolerances. Critical process windows must be established, particularly regarding the resolution limits of hydrophobic barriers and thermal processing uniformity. Furthermore, mitigating the effects of environmental humidity and substrate heterogeneity is essential to maintain consistent flow rates and reliability during mass production [168]. Quantifiable manufacturing metrics such as relative standard deviation of channel equivalent width and contact angle RSD must align with clinical and regulatory requirements for intra- and inter-batch consistency standards. Implementation of roll-to-roll manufacturing processes and digital laser or thermal transfer technologies offers promising pathways to enhance batch uniformity while maintaining cost-effectiveness. Surface engineering methods, including iCVD and plasma treatment, demonstrate potential for stabilizing wetting characteristics and extending shelf life, yet standardized protocols for quality control remain underdeveloped [73,169].

While cost-effective and intuitive, paper-based colorimetric detection faces significant challenges in low-copy nucleic acid testing and complex matrices such as serum and saliva. Background color variations, non-specific adsorption, and diffusion dilution effects compromise signal-to-noise ratio and limit detection sensitivity. Current mitigation strategies include: coupling with CRISPR/Cas trans-cleavage amplification systems to enhance sensitivity [104,170]; implementing electrochemical readout methods to reduce optical background interference [171,172]; integrating preconcentration/extraction zones to increase target density per unit volume [173]; and developing standardized smartphone imaging protocols and machine learning-based regression algorithms to minimize readout variance. However, establishing reference color standards, internal reference channels, and image calibration algorithms requires community consensus and standardized reporting protocols to address reproducibility gaps.

Two-dimensional and three-dimensional structures combined with origami layouts inherently support spatial parallelization and sequential processing, but multiplexed target detection imposes strict requirements for cross-reaction prevention, fluid crosstalk mitigation, and timing control. Recent advances in multiplexed nucleic acid detection utilizing spatial encoding and CRISPR systems indicate that paper-based platforms can benefit from array-based and encoding strategies, including spatial encoding and barcode-guided readout. On-chip implementation of separated hydrophobic walls, delay channels, and programmable dissolvable or wax valves enables timing programmability, while smartphone-based AI interpretation facilitates interpretable multiparameter diagnostic readouts in field and primary care settings. However, the complexity of fluid routing and timing control in multiplexed systems presents ongoing engineering challenges requiring systematic optimization.

Quantitative analysis in μPADs is significantly affected by illumination conditions, camera sensor variations, and color differences in paper substrates between manufacturers and batches. The absence of standardized reference materials and calibration procedures leads to reproducibility issues of different conclusions with the same method, undermining clinical validation efforts. Addressing these challenges requires developing internal reference channels, standardized color cards, and robust image calibration algorithms capable of compensating for device-specific variations. Machine learning approaches show promise in reducing measurement variance, but their implementation requires extensive validation across diverse operating conditions and user populations.

The ultimate success of μPADs in POC applications depends on the seamless integration of pump-free microfluidics with visual output platforms while maintaining user-friendliness and meeting quantitative and regulatory requirements. Engineering approaches should focus on capillary-driven fluid control mechanisms, including geometric delays, sugar bridges or wax valves, and membrane valves, combined with low-cost visualization methods such as colorimetric edge lighting and smartphone readout capabilities. Front-end manufacturing improvements through R2R or digital laser or thermal transfer processes must be combined with midstream surface engineering for wettability stability and shelf life, while backend solutions require integrating internal references, standardized color cards, and algorithm-based quantitative comparability.

To systematically address these challenges, the field requires interdisciplinary collaboration spanning materials science, mechanical engineering, and information technology, complemented by extensive practical validation in home-use scenarios. Key research priorities include establishing statistical metrics and control limits for manufacturing processes with inline quality control evidence chains; developing standardized protocols for cross-device illumination and camera calibration, providing comprehensive raw image datasets; optimizing signal enhancement mechanisms through CRISPR, enzymatic or nanozymatic, and electrochemical methods, quantifying amplification factors and realistic LOD assessments in paper-based backgrounds; implementing spatial multiplexing strategies with crosstalk avoidance, fault-tolerant design, and automated interpretation algorithms; and defining user-teachable operation procedures with error tolerance and human–computer interfaces for smartphone-based home testing applications. Successful resolution of these challenges will position μPADs as transformative technologies for global health, enabling accessible, quantitative, and reliable point-of-care diagnostics at home.

## Figures and Tables

**Table 1:** The comparison between two major fabrication methods.

Category	Tecnical Method	Basic Principle	Typical Resolution	Cost and Scalability	Core Advantages	Main Limitations	Reference
(1) Automated Scalable Production	Wax printing	Print hydrophobic wax ink on paper, heat to penetrate fibers to form fluid barriers	~500 µm	Low Scalability: High	Extremely simple operation Minimal material costs Suitable for rapid prototyping and batch production	Limited resolution Thermal penetration difficult to control precisely Commercial wax printers being discontinued	[38]
(2) Automated Scalable Production	Roll-to-roll processing	Automated patterning on continuous flexible paper rolls (often integrating wax printing or screen printing), achieving continuous production	Depends on core process	High (equipment)Scalability: Extremely High	True industrial-scale production Minimized unit cost at scale Continuous manufacturing	Huge initial equipment investment Complex process debugging Suitable only for mature products	[39]
(3) Automated Scalable Production	Laser etching	Use high-energy laser beams to burn or cut paper, physically removing material to form channels and structures	~100 µm	Medium Scalability: Medium-High	High precision No chemical reagents needed Rapid fabrication of complex 2D/3D structures Easy automation	Edges may be rough and carbonized Potential impact on fluid behavior High-speed equipment costs high	[40]
(4) Automated Scalable Production	Screen printing	Use screen templates to press hydrophobic polymers or functional inks (conductive ink, enzyme ink) onto paper surfaces	~100–200 µm	Medium Scalability: High	Low unit cost for batch production Can integrate functional elements (electrodes) in one step Mature industrial process	High initial plate making cost Resolution limited by screen mesh Not suitable for rapid prototyping	[41]
(5) Refined Customization Methods	Photolithography	Use photomask and UV light to cure photoresist, after development form high-precision hydrophobic barriers	<100 µm	High Scalability: Low	Highest resolution Most complex microfluidic structures achievable Gold standard for laboratory research	High cost Requires cleanroom environment Chemical residues may affect biocompatibility	[42]
(6) Refined Customization Methods	Chemical Vapor Deposition (CVD)	Gaseous monomers under mask protection selectively polymerize on paper fiber surface to form hydrophobic coating	~50–200 µm (limited by creep effect)	High Scalability: Low	Uniform and stable coating Excellent chemical resistance and hydrophobicity High-performance fluid control	Expensive equipment Complex process Gas-phase “creep effect” blurs pattern edges	[41]
(7) Refined Customization Methods	Plasma treatment	Use plasma under mask protection to selectively alter paper surface chemical properties for micro-scale hydrophilic/hydrophobic patterning	~10–50 µm (with physical masks)	Medium Scalability: Low	Precise control of surface wettability Uniform treatment Enhanced biomolecule immobilization efficiency	Requires specialized equipment Treatment effect may decay over time High-precision requires tight mask fitting	[41]

**Table 2:** Comparison of μPAD-based Nucleic Acid Extraction Methods.

Method	Material	Extraction Efficiency	Sample Type	Cost (USD)	Key Features	Reference
Electrophoretic μPADs	Glass Microfiber (Whatman GF/F)	~80–90%	Water, Cell Lysate	<1	Electrophoretic separation, multi-layer DNA movement	[93]
UV-Ozone Treated Glass Fiber	Glass Fiber	>90%	Whole Blood	~1	2.5× improved recovery, LAMP integration	[36]
Chitosan-Modified Glass Fiber	Chitosan-Modified Fusion 5	95–98%	Genomic DNA, λ-DNA	~1–2	In situ PCR, high efficiency	[94]

**Table 3:** The list of home-based μPADs diagnostics.

Pathogen	Target Gene	Isothermal Amplification	Detection Limit	Analysis Time	Sample	Remark	Structure	Estimated Cost (USD)	References
SARS-CoV-2	N, E, S genes	RT-LAMP	10–310 copies/mL	~120 min	Wastewater	Semi-quantitative detection on paper device; under laboratory-controlled conditions	Multi-layer paper chip	5–15	[101]
SARS-CoV-2	N gene	RPA	1000 copies/mL	~20–40 min	NP swab	All-in-one CRISPR visual readout; under PoC settings	Paper/crispR integrated	<5	[102]
SARS-CoV-2	ORF1a	RT-RPA	10 copies/µL	50 min	Nasopharyngeal/saliva	Extraction-free, in-tube fluorescence; smartphone quantification; under PoC settings	Paper-compatible tube/sealed readout	<5	[103]
SARS-CoV-2	E/N genes	RT-LAMP	10 copies/µL	30–40 min	NP/oropharyngeal	Dual-gene lateral flow (visual); under PoC settings	Lateral-flow paper cassette	<5	[104]
Ebola/Lassa	EBOV L; LASV L and S	RPA	~10 copies/µL	<1 h	Blood/urine/saliva	HUDSON inactivation demonstrated; field-deployable; under PoC settings	Sealed fluorescence/LFA readout	<5	[30]
Malaria	*Plasmodium* spp.: 18S rRNA	LAMP	5 parasites/µL	55 min	Finger-prick blood	field-tested in Uganda village clinics; under PoC settings	wax patterned origami folding structure	<5	[99]
Zika/Chikungunya/Dengue	ZIKV: NS5; CHIKV: E1; DENV: 3′-UTR/C-prM	RT-LAMP	10 PFU/mL	45 min	Serum	QUASR RT-LAMP with smartphone detection; closed-tube to avoid contamination; under PoC settings	Portable LAMP box + smartphone readout	<5	[105]
*E. coli*/*Salmonella*/*S. aureus*	rfbE, invA, nuc/femA	LAMP	12 CFU/mL	<1 h	Food/water	Paper-embedded microchip; fluorescence/color readout; under PoC settings	Paper-embedded microchip	<5	[106]
Ostreopsis cf. ovata	rDNA	RPA	0.06 pM action	95 min	Environmental DNA	Three-dimensional μPADs with gold-thread electrodes; under laboratory conditions	3D μPADs	5–15	[82]
Food quality	antioxidants		0.005 µM	<10 min	Honey brown sugar	Nanozyme colorimetric array; multivariate analysis; under PoC settings		<5	[107]
Prostate cancer	PCA3 mRNA		0.34 fg/µL	75 min	Cell RNA/urine	3D-printed μPADs; smartphone imaging; calcein colorimetry; under laboratory conditions	3D-printed μPADs	<5	[108]
Cancer	miR-21	RT-LAMP	4.1 pM	~30 min	Urine	Y-shaped μPADs; enzyme-mimetic nanoclusters; under PoC settings	Y-shaped μPADs	<5	[109]
*C. trachomatis*	Cryptic plasmid	tHDA	10^4^ cells/mL	<50 min	Synthetic urine	Paper-based extraction in situ amplification; under laboratory conditions	Folded paper in pipette tip	<5	[110,110]
HIV	HIV-1 RNA	RT-RPA	5000 copies/mL serum	45 min	Human serum	Paper-based ITP RNA extraction; duplexed detection with MS2 control; under PoC settings	2D paper network	<5	[111]
HIV	HIV-1 RNA	RT-LAMP	30 copies/mL serum	35 min	Human serum/plasma	Handheld device with ball-valve fluidics; real-time fluorescence detection; under PoC settings	3D-printed handheld device	5–15	[112]
HPV	High-risk HPV DNA	Hybrid capture	6.6 × 10^4^ copies/mL	60 min	Cervical cells	2D paper network for hybrid capture; colorimetric detection; under laboratory conditions.	Layered nitrocellulose/glass fiber	<5	[113]

## Data Availability

No new data were created or analyzed in this study. Data sharing is not applicable to this article.
